# Ultraviolet C Irradiation Induces Different Expression of Cyclooxygenase 2 in NIH 3T3 Cells and A431 Cells: The Roles of COX-2 Are Different in Various Cell Lines

**DOI:** 10.3390/ijms13044351

**Published:** 2012-04-05

**Authors:** Ming-Hong Tai, Chien-Hui Weng, Dir-Pu Mon, Chun-Yi Hu, Ming-Hsiu Wu

**Affiliations:** 1 Institute of Biomedical Sciences, National Sun Yat-Sen University, Kaohsiung 80424, Taiwan; E-Mail: minghongtai@gmail.com; 2 Department of Biological Sciences, National Sun Yat-Sen University, Kaohsiung 80424, Taiwan; E-Mail: wch5471@yahoo.com.tw; 3 Department of Nutrition and Health Science, Fooyin University, Kaohsiung 83102, Taiwan; E-Mails: pumpmon@gmail.com (D.-P.M.); mt121@mail.fy.edu.tw (C.-Y.H.); 4 Research Center of Health Food, Fooyin University, Kaohsiung 83102, Taiwan

**Keywords:** ultraviolet C (UVC), cyclooxygenases-2 (COX-2), prostaglandin E2 (PGE2), NIH 3T3 cells, A431 cells

## Abstract

Ultraviolet C (UVC) is a DNA damage inducer, and 20 J/m^2^ of UVC irradiation caused cell growth inhibition and induced cell death after exposure for 24–36 h. The growth of NIH 3T3 cells was significantly suppressed at 24 h after UVC irradiation whereas the proliferation of A431 cells was inhibited until 36 h after UVC irradiation. UVC irradiation increased COX-2 expression and such up-regulation reached a maximum during 3–6 h in NIH 3T3 cells. In contrast, UVC-induced COX-2 reached a maximum after 24–36 h in A431 cells. Measuring prostaglandin E2 (PGE2) level showed a biphasic profile that PGE2 release was rapidly elevated in 1–12 h after UVC irradiation and increased again at 24 h in both cell lines. Treatment with the selective COX-2 inhibitor, SC-791, during maximum expression of COX-2 induction, attenuated the UVC induced-growth inhibition in NIH 3T3 cells. In contrast, SC-791 treatment after UVC irradiation enhanced death of A431 cells. These data showed that the patterns of UVC-induced PGE2 secretion from NIH 3T3 cells and A431 cells were similar despite the differential profile in UVC-induced COX-2 up-regulation. Besides, COX-2 might play different roles in cellular response to UVC irradiation in various cell lines.

## 1. Introduction

Ultraviolet (UV) light includes UVA (wavelength, 320–400 nm), UVB (wavelength, 290–320 nm), and UVC (wavelength, 200–290 nm) [1]. UVA light is poorly absorbed by most biomolecules, but produces active oxygen intermediates and results in cytotoxicity by generating free radicals. UVB is directly absorbed by nucleic acids and generates reactive oxygen species, inducing DNA damage and oxidative stress. UVC is also strongly absorbed by nucleic acids, leading to DNA damage; it causes either cell death or mutation, which is considered to be one of the initial steps of carcinogenesis [1,2]. UV irradiation not only causes damage of cellular components, but also induces specific cellular reactions. For example, UV light induces expression of activator protein-1 (AP-1), p53, nucleophosmin/B23 (NPM/B23) and cyclooxygenases-2 (COX-2) [1,3–7]. Cyclooxygenases (COXs) catalyze the first and rate-limiting step in the conversion of arachidonic acid into prostaglandin (PG), prostacyclins, and thromboxanes [8]. There are two isoforms of COXs in cells: COX-1 is constitutively expressed in most tissues and its PG products are involved in maintaining tissue homeostasis, while COX-2 is normally not expressed in most tissues and is highly inducible by many stimuli, including cytokines, growth factors, and UV light [4,9]. PGs produced by COX-2 contribute to pathophysiological functions, such as inflammation, pain, fever, wound repair, angiogenesis, vasodilatation and increased vascular permeability [8]. Prostaglandin E2 (PGE2) is the major PG produced by the skin after UVB irradiation, and serves as a mediator of UVB-induced skin alterations [4,10]. The increased production of PGE2 following UVB exposure correlates with increased COX-2 activity as well as the induction of COX-2 mRNA [4,11]. Numerous reports indicate that COX-2 plays an important role in inflammation, cell growth, apoptosis, and carcinogenesis [4,8]. Use of selective COX-2 inhibitors and genetic manipulation of COX-2 expression demonstrated that UVB induction of COX-2 in the skin contributes to the induction of epidermal hyperplasia, inflammation, edema, and counteracts the induction of apoptosis after UVB exposure [4,8,10].

Although UVC is blocked by the atmosphere, people may be irradiated by UVC from germicidal lamps. UVB causes not only DNA damage, but also induces oxidative stress [1,2]. Because UVC merely induces DNA damage, UVC is usually employed as a study tool to investigate the cellular response to DNA damage [5–7,12–14]. UVC closely resembles UVB in gene activation, but UVC causes a higher degree of DNA damage than UVB [1,15]. Induction of apoptosis by UVB and UVC irradiation is initiated by DNA damage [1], and the application of laser-pulsed UV light in the treatment of human tumors has been suggested [14]. Induction of apoptosis pathways includes extrinsic and intrinsic pathways to activiate different caspase casades: the extrinsic pathway involves the binding of death ligands, and the intrinsic pathway is the translocation of cytochrome C release from mitrochondria to cytosol [16]. However, it is reported that distinct apoptotic signalling caused by UVB and UVC: UVB induces apoptosis by both extrinsic and intrinsic pathways, while UVC elicits apoptosis only via the intrinsic pathway [17].

NIH 3T3 cells are derived from embryonic cells and are considered as normal cells [12], while A431 cells are derived from epidermoid carcinoma and are skin cancer cells [14]. Both cell lines are usually applied on the cell biology study of UV irradiation [5,6,12,14]. Many studies have reported the induction of COX-2 by UVA or UVB, but very few papers report the role of COX-2 in cellular response to UVC or DNA damage. In this study, we characterized the induction of COX-2 and PGE2 following UVC exposure of NIH 3T3 and A431cells. We found that UVC-induced patterns of PGE2 secretions from NIH 3T3 cells and A431 cells were similar, while the UVC-induced profiles of COX-2 up-regulation were different in these two cell lines. Although many reports indicate that induction of PGE2 is correlated with the activities of COX-2 [3,11,18,19], our data suggested that the level of PGE2 is not suitable for representing the activity of COX-2 after UVC treatment in NIH 3T3 and A431 cells. COX-2 might play different roles in cellular response to UVC irradiation in various cell lines.

## 2. Results and Discussion

### 2.1. The COX-2 Inhibitor (SC-791) Attenuated UVC-Induced Apoptosis in NIH 3T3 Cells, but not in A431 Cells

**Figure 1:**
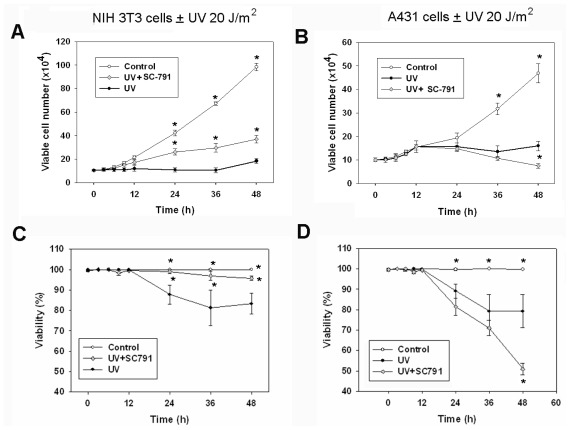

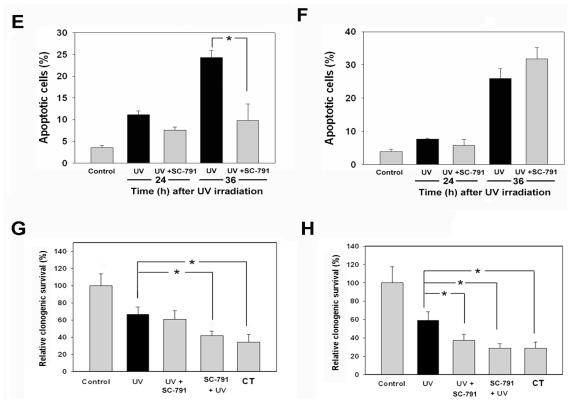
Effect of COX-2 inhibitor on cell growth and survival after UVC irradiation in NIH 3T3 cells and A431 cells. NIH 3T3 cells and A431 cells were grown in 12-well plates overnight before treatment. Control: cells were mock-treated, and the same procedure was followed without irradiation. UV: cells were irradiated with UVC at 20 J/m^2^. UV + SC-791: cells were irradiated with UVC at 20 J/m^2^, and then the COX-2 inhibitor SC-791 was added at 2 h after UVC irradiation, SC-791 was removed by change of medium at 8 h after irradiation, and the cells were briefly treated with SC-791 for 6 h in total. SC-791 + UV, 30 min before UVC irradiation, cells were pre-treated with COX-2 inhibitor SC-791 (10 μM), and then cells were irradiated with UVC at 20 J/m^2^. CT (co-treatment): cells were irradiated with UVC at 20 J/m^2^, and then were immediately treated with 10 μM of SC-791. Viable and non-viable cells were determined by the trypan blue exclusion method, and the percentage of apoptotic cells was measured by an image cytometer. (**A**) and (**B**): Number of viable cells following exposure of UVC in NIH 3T3 cells and A431 cells; (**C**) and (**D**): Cell viability percentages subsequent to exposure of UVC in NIH 3T3 cells and A431 cells; (**E**) and (**F**): The percentage of apoptotic cells after UVC exposure in NIH 3T3 cells and A431 cells; (**G**) and (**H**): Relative clonogenic survival following UVC irradiation in NIH 3T3 cells and A431 cells. Points and bars, means of triplicates ± SD. * *P* < 0.05, as compared with cells which were treated with the same dose of UVC irradiation for the same times.

The growth of NIH 3T3 cells was significantly reduced by UVC irradiation (20 J/m^2^) within 24 h and sustained for at least 48 h (Figure 1A). The growth of A431 cells was not significantly inhibited by UVC until 36 h after irradiation (Figure 1B). To distinguish viable and non-viable cells, we used trypan blue to stain non-viable cells. The trypan blue positive cells were non-viable, or dead cells significantly appeared at 24, 36 and 48 h after UVC exposure in both cell lines, and the percentage of cell viability also reduced in parallel (Figure 1C,D). Induction of cell death by UVC required longer times, and such delayed cell death (>20 h) was also observed after UVB treatment, as noted in other reports [2,5]. In this study, 20 J/m^2^ UVC irradiation caused a slow progression of apoptosis in NIH 3T3 and A431 cell lines, while very high doses of UVC irradiation (>100 J/m^2^) resulted in atypical cell death, which is not an optimal condition to study cellular response to UVC-induced DNA damage (data not shown).

To study the role of COX-2 in cellular response to UVC irradiation, the selective COX-2 inhibitor SC-791 was applied to test the growth and viability of the irradiated cells. The cell growth and clonogenic survival assays revealed that the SC-791 experimental condition (10 μM for 6 h) was not toxic to NIH 3T3 and A431 cells (data not shown). In NIH 3T3 cells, exposure to SC-791 (10 μM) during 2–8 h after UVC irradiation significantly restored the cellular growth (Figure 1A). Short exposure of SC-791 at this period also attenuated UVC-induced cell death (Figure 1C) and apoptosis (Figure 1E) in NIH 3T3 cells. However, SC-791 application did not confer protection to UVC-irradiated A431 cells (Figure 1B,D,F). The effect of the COX-2 inhibitor SC-791 on UVC-irradiated NIH 3T3 cells was time specific, prior application of SC-791 before irradiation did not confer protection against UVC-induced growth inhibition or apoptosis. The clonogenic survival assay was employed to assay the long-term survival of cells after UVC irradiation, and the number of colonies was determined at the 10th day following exposure to UVC irradiation (Figure 1G,H). Such treatment of UVC-irradiated NIH 3T3 and A431 cells by SC-791 did not increase long-term cell survival, and pretreatment or co-treatment of SC-791 with UVC irradiation at other time points contrarily enhanced the cytotoxicity of UVC irradiation (Figure 1G,H). In A431 cells, exposure of SC-791 during 20–36 h after UVC irradiation did not prevent the UVC-induced death (data not shown). These data implied that up-regulation of COX-2 might play a role involving growth inhibition in cellular response to UVC in NIH 3T3 cells.

### 2.2. UVC-Induced Different Distributions of Cell Cycle in NIH 3T3 Cells and A431 Cells

**Figure 2:**
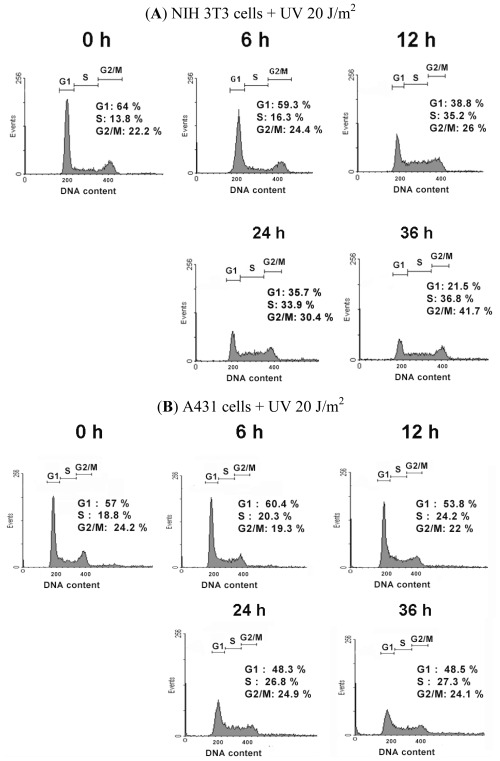

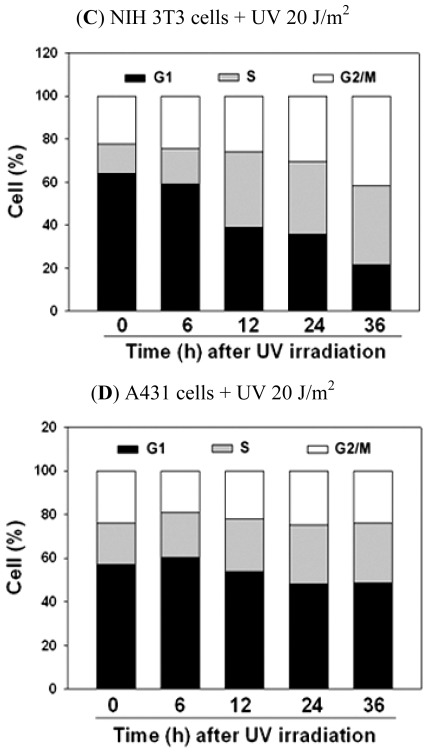
Effect of UVC on cell cycle progression in NIH 3T3 cells and A431 cells. (**A**) and (**B**): Flow cytometry analysis of cell cycle distribution in NIH 3T3 cells and A431 cells at various time intervals after UVC irradiation. The percentages of cells at specific phases were indicated; (**C**) and (**D**): Quantitative analysis of cell cycle distribution in NIH 3T3 cells and A431 cells at various time intervals after UVC irradiation. The Figure 2A,B is representative of results obtained from three independent experiments. The data of Figure 2C,D is the average from three independent experiments, and the raw data and statistical analysis are displayed in Table 1.

Because UVC-induced growth inhibition occurred earlier in NIH 3T3 cells than in A431 cells, we assayed cell cycle distributions and compared their differences after UVC irradiation. Cytometric DNA profile data showed that UVC irradiation reduced the population of G1 cells, and caused S and G2/M phase arrest of NIH 3T3 cells (Figure 2A and Table 1). In UVC-irradiated A431 cells, a similar trend of cell cycle progression was observed, but was not apparent as in UVC-irradiated NIH 3T3 cells (Figure 2B and Table 1). The first 12 h after UVC irradiation, the growth of A431 cells were not completely inhibited (Figure 1B), and the distributions of the cell cycle were slightly affected by UVC (Figure 2B and Table 1). Figure 2C,D showed the effect of UVC on the progression of cell cycle by stacked bar chart. Figure 2C and Table 1 indicated that the population of NIH 3T3 cells gradually decreased in G1 phase cells, and increased in the S and G2/M phase cells following exposure to UVC irradiation. However, in A431 cells, the distributions of cell cycle from most of the time points were not statistically significant: different when compared to that of control cells, except G1 phase at 6 h and S phase at 24 and 36 h. The G1 phase decreased slightly at 6 h, while the S phase increased at 24 and 36 h after UVC irradiation (Table 1). The statistical analysis from 36 h following irradiation indicated that UVC significantly reduced the population of the G1 phase, and arrested the cell cycle at the S and G2/M phase in NIH 3T3 cells. However, UVC only arrested the cell cycle at the S phase in A431 cells. This seemed be due to the fact that A431 cells are cancer cells with mutant p53 and defective for the G1/S checkpoint [20], thus lack immediate mechanisms for blocking the cell cycle at the G2/M phase. It is reported that UVC-damaged DNA causes growth inhibition and arrests cell cycle at the S and G2/M phases [21,22], which is consistent with our results in NIH 3T3 cells.

### 2.3. The Kinetics of COX-2 Protein Induction after UV Irradiation Were Different in Various Cell Lines

**Figure 3:**
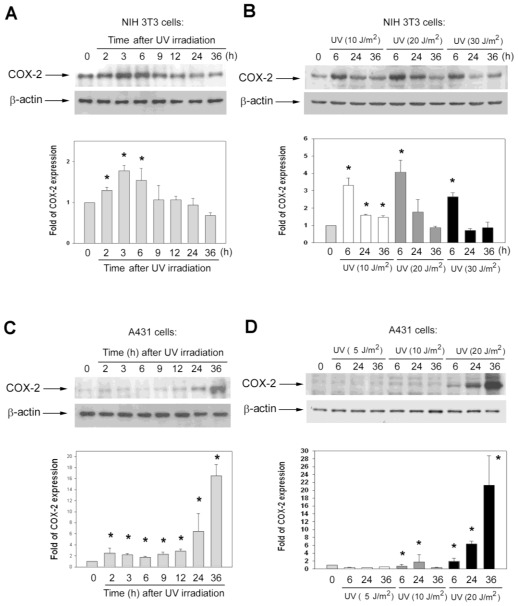
The kinetics of COX-2 protein induction after UV irradiation in NIH 3T3 cells and A431 cells. (**A**) and (**C**): Immunoblot analysis of COX-2 expression after 20 J/m^2^ of UVC irradiation in NIH 3T3 cells and A431 cells; (**B**) and (**D**): The dose- and time-dependent effect of UVC irradiation on COX-2 expression in NIH 3T3 cells and A431 cells. Cells were exposed to 5, 10, 20, or 30 J/m^2^ of UVC irradiation and harvested at the indicated times. Equal amounts of protein were separated with 10% SDS-PAGE and blotted onto PVDF membranes. β-actin was used as a control for equal loading. Histogram: quantifications of COX-2 and β-actin immuno-band intensities were determined by densitometric scanning, and the values of COX-2 were normalized with respect to the intensities of β-actin. Bars, means of triplicate ± SD. * *P* < 0.05, as compared with the corresponding values for COX-2 which were not treated with UVC (0 h; control group).

Immunoblot analysis was performed to investigate the effects of UVC irradiation on the expression of COX-2. In NIH 3T3 cells, UVC irradiation rapidly elevated COX-2 levels; COX-2 protein measurably increased at 2 h after exposure and reached a maximum during 3–6 h (Figure 3A). This result resembles another study reporting that COX-2 is evidently induced in embryo fibroblasts by UVC [13]. In A431 cells, COX-2 levels increased slowly after exposure of UVC; COX-2 protein was not induced by UVC irradiation until cell death occurred (during 24–36 h post-irradiation) (Figure 3C). It is reported that protein levels of COX-2 is undetectable in most normal epithelial tissues, and up-regulation of COX-2 is believed to confer resistance to apoptosis [23]. To clarify the role of COX-2 which was induced during 24–36 h post-irradiation in A431 cells, we detected the variation of COX-2 after exposure to different doses of UVC at 6, 24, and 36 h in NIH 3T3 cells and A431 cells (Figure 3B,D). Low dose (5 and 10 J/m^2^) of UVC irradiation inhibited cell growth and induced low levels of cell death, with most cells surviving after irradiation (data not shown). In NIH 3T3 cells, the kinetic profiles of COX-2 in response to different doses of UVC were similar, even the dose of UVC was raised to 30 J/m^2^ (Figure 3B). However, in A431 cells, the kinetic profiles of COX-2 in response to different doses of UVC were different: COX-2 was still elevated at 36 h after exposure to UVC at 20 J/m^2^, but did not evidently induce at 36 h after exposure to 5 and 10 J/m^2^ of UVC (Figure 3B). In A431 cells, low doses of UVC (5 and 10 J/m^2^) only caused low percentages of dead cells (data not shown), and 20 J/m^2^ of UVC induced a 26% appearance of apoptotic cells at 36 h (Figure 1D) and finally killed ~60% cells, analyzed by relative clonogenic survival assay (Figure 1F). Therefore, we suggested the role of COX-2 that was induced in A431 cells during 24–36 h post-irradiation was associated with UVC-induced cell death.

### 2.4. The Kinetics of PGE2 Induction after UVC Irradiation Was Similar in Various Cell Lines

**Figure 4:**
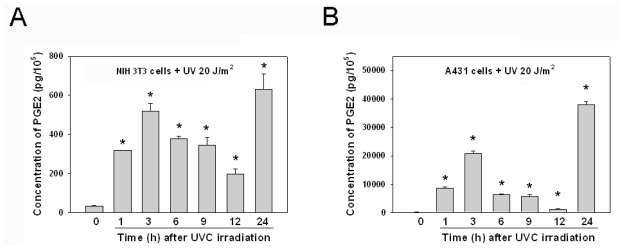
Effect of UVC irradiation on PGE2 production in NIH 3T3 cells and A431 cells. (**A**) Induction of PGE2 after UVC irradiation in NIH 3T3 cells; (**B**) Induction of PGE2 after UVC irradiation in A431 cells. The concentrations of PGE2 secreted from UVC-irradiated cells were determined by competitive enzyme-linked immunosorbent assay kit with an ELISA reader. Data were mean ± SD of triplicates. * *P* < 0.05, as compared with the concentrations of PGE2 secreted from control cells at initial time (0 h).

While COX-2 protein was elevated after UVC irradiation in NIH 3T3 cells (Figure 3A), the concentrations of PGE2 were measured in parallel in cell-free culture media collected from UVC-irradiated cells at indicated times (Figure 4A). Because the culture media were changed to new media at 30 min before harvest, the concentrations of PGE2 in the media were newly secreted from UVC irradiated cells. PGE2 production was rapidly induced upon UVC irradiation, giving two PGE2 peaks. The first PGE2 peak nearly correlated with COX-2 protein levels at 1–12 h post-irradiation, but there was no correlation between PGE2 and COX-2 at 24 h post-irradiation. The second intense PGE2 peak occurred 24 h after UVC irradiation, while COX-2 protein levels decreased at the same time (Figure 4A). In A431 cells, the PGE2 level was also rapidly induced, and there were also two PGE2 peaks following UVC irradiation: the first peak appeared 1–12 h post-irradiation, and the second intense peak occurred at 24 h following UVC irradiation (Figure 4B). The pattern of PGE2 induction after UVC irradiation was similar in NIH 3T3 cells and A431 cells, but the pattern of COX-2 protein induced after UVC irradiation was different in the two cell lines (Figures 3 and 4). Because brief exposure of 10 μM SC-791 during 2–8 h after UVC irradiation attenuated UVC-induced growth inhibition in NIH 3T3 cells (Figure 1A), the effect of SC-791 on the concentrations of PGE2 were also measured following exposure of UVC. We also found the UVC-induced PGE2 secretion was blocked during SC-791 treatment (10 μM for 2–8 h after UVC irradiation) in both cell lines. After removal of SC-791 the secreted PGE2 level from UVC-irradiated cells were similar (data not shown), suggesting the transient inhibitory effect of COX-2 by SC-791.

### 2.5. Discussion

Our data showed that the pattern of PGE2 induction by UVC irradiation was similar in NIH 3T3 cells and A431 cells (Figure 4), but the pattern of COX-2 protein induced by UVC irradiation differed between the two cell lines (Figure 3). Because COX-2 is an inducible enzyme for production of PGs, the induction of PGE2 is usually considered to be an indirect indicator of COX-2 activity. Several studies reported the concentrations of PGE2 to be an indicative of the activity of COX-2 [3,19]. However, our data suggest that the PGE2 is not absolutely suitable for representing the activity of COX-2 after UVC irradiation. Our data showed that the expression of COX-2 protein was not correlated with the induction of PGE2 after UVC irradiation in the two cell lines (Figures 3 and 4). PGE2 can be produced by COX-2 and COX-1. COX-1 is constitutively expressed, while COX-2 is inducible by certain stimuli [8]. Thus, additional mechanisms may be involved in the conversion of PGE2, provoked by UVC in NIH 3T3 and A431 cells. For example, a recent study reported that down-regulation of 15-hydroxyprostaglandin dehydrogenase, an enzyme involving degradation of PGE2, contributes to the elevated levels of PGs in skin following UV exposure [24]. Future studies are warranted to elucidate whether such UV-reduced PGE2 catabolism exists in UVC-irradiated NIH 3T3 and A431 cells.

In A431 cells, the level of COX-2 is relatively low before UVC irradiation, and this phenomenon is correlated with other reports that expression of COX-2 is low or undetectable in most tissues [4,9]. In contrast, constitutive COX2 expression was found in NIH 3T3 cells before UVC irradiation (Figure 3). Because NIH 3T3 cells are embryo fibroblasts, our finding is consistent with previous studies that embryonic cells constitutively express COX-2 and produce PGE2 to prevent cells from apoptosis [19]. It has been reported that the mechanism of COX-2 expression in cellular response to UVC causes the UVC-induced transcription activation of COX-2 to be delayed in mouse embryo fibroblasts if eukaryotic initiation factor 2 (elF2) is without phosphorylation [13]. Different cells might be expressed by various degrees of phosphorylation of elF2, and thus may affect the level of COX-2 expression. Moreover, there are several distinct expression of genes between NIH 3T3 cells and A431 cells, which might affect COX-2 expression in response to UVC, including expression of normal p53, but no expression of epidermal growth factor receptor (EGFR) in NIH 3T3 cells, and over-expression of mutant p53 and EGFR in A431 cells [14,20,25]. EGFR localizes on the cell surface and is a receptor of tyrosine kinase, related to malignant disease, and is one of the pathways activated by UV irradiation [1].

There were two peaks of PGE2 induction subsequent to UVC exposure in NIH 3T3 cells and A431 cells. The first peak of PGE2 was rapidly induced and was lower compared to the second peak of PGE2 (Figure 4). Because UVC-raised COX-2 nearly correlated with the first peak of PGE2 in NIH 3T3 cells, treatment with the selective COX-2 inhibitor SC791 during this period attenuated the UVC induced-growth inhibition and apoptosis in NIH 3T3 cells (Figure 1A,C,E). UVC-damaged DNA causes growth inhibition and cell cycle arrest for DNA repair [1,6,21,22], and therefore we speculated that UVC-elevated COX-2 of NIH 3T3 cells could play a role involving the inhibition of cell growth or anti-stress in response to UV irradiation. We also observed that COX-2 of A431 cells was not immediately induced after exposure of UVC (Figure 3). Hence, we speculated this to be the reason why treatment with the COX-2 inhibitor did not restore UVC-inhibited growth in A431 cells (Figure 1B). COX-2 is an early response gene, which can be stimulated rapidly and transiently by cytokines, growth factors, and UV irradiation [26]. Evidently, COX-2 is not an immediate early gene in response to UVC in A431 cells. Over-expression of COX-2 inhibits UVB-induced apoptosis, and activates anti-apoptotic signaling by PGE2 receptors [18]. Although COX-2 contributes to anti-apoptosis, tumor growth and metastasis [4,8,26], over-expression of COX-2 decreases proliferation and increases apoptosis in certain cell lines [27,28]. The effects of COX-2 over-expression could be cell-type dependent [23,29], and our studies suggest that COX-2 may have different roles in pathophysiological conditions.

In this study, the first UVC-induced COX-2 expression and PGE2 release occurs within 2 h after UVC irradiation in NIH 3T3 cells. Such COX-2 up-regulation might be associated with growth inhibition responding to cellular stress, since COX-2 inhibition at this stage rescues cells from death. Contrariwise, the first UVC-induced COX-2 up-regulation takes places later in A431 cells when cells undergo apoptosis. This may represent the cellular attempt to counteract cell death. Interestingly, the second peak of UVC-induced PGE2 was the highest in both cell lines, when cell death events were most active during this period (Figures 1 and 4). Thus, it is speculated this second PGE2 peak could be involved in summoning inflammatory cells to clear cell debris and be responsible for sunburn and UVC-induced erythema and inflammation, which usually occurs at 24 h after UV exposure [30,31].

## 3. Experimental Section

### 3.1. Reagents and Antibodies

All chemicals were purchased from Sigma (St. Louis, MO, USA), except where otherwise indicated. Anti-COX-2 polyclonal antibody (Cayman, Ann Arbor, MI, USA) and anti-β-actin monoclonal antibody were purchased from Novus (Littleton, CO, USA), and the secondary antibody, horseradish peroxidase-conjugated goat anti-mouse IgG antibody, was purchased from Jackson ImmunoResearch (Baltimore, PA, USA). The selective COX-2 inhibitor, SC-791, 4-[(5-Difluoromethyl-3-phenyl)-4-isoxazolyl] benzenesulfonamide, was purchased from Calbiochem (Darmstadt, Germany), which is a cell-permeable compound that acts as a potent and highly selective inhibitor of COX-2 both *in vitro* (IC_50_ of 4 nM) and *in vivo* [25,26]. It was also reported that even 24 h treatment of 10 μM of SC-791 did not cause significant cell death in MCF-7 human breast cancer cells [32].

### 3.2. Cell Culture and UVC Treatments

Cells were cultured in Dulbecco's modified Eagle's medium (Life Technologies, Rockville, MD, USA) supplemented with 10% heat-inactivated serum (Hyclone, UT, USA)(bovine calf serum for NIH 3T3 cells; fetal bovine serum for A431 cells), 0.5% antibiotics and 3.7 g/L sodium bicarbonate in a 5% CO_2_ humidified incubator at 37 °C. Numbers of viable and dead cells were determined by trypan blue exclusion and counted with a hemocytometer. UVC treatments were performed with a CL-1000 Ultraviolet Crosslinker equipped with UVC lamps (UVP, Upland, CA, USA) to produce indicated doses of UVC irradiation at 254 nm; the instrument is equipped with a sensor to automatically monitor and control the exposure time and strength of UVC. Before UVC irradiation, the culture medium was removed, and then original medium was added to cells. Cells were harvested and counted at indicated times.

### 3.3. Cell Growth Curve and Clonogenic Survival Assay

NIH 3T3 cells and A431 cells were grown in 12-well plates over night, and then were irradiated with 20 J/m^2^ of UVC. The cells were harvested at indicated times by trypsinization, and the numbers of viable cells determined by trypan blue exclusion were counted in a hemocytometer. The relative clonogenic survival (relative cell survival assay) cells were cultured in 6-well plates, and appropriate numbers of cells were seeded in triplicate wells to produce at least 30 colonies per well. For NIH 3T3 cells, 1 × 10^3^ cells per well were seeded, and for A431 cells, 2 × 10^3^ cells per well were seeded. UVC irradiation of cells was performed at 24 h after seeding. Ten days after irradiation, clones were stained with 0.5% crystal violet (in 70% methanol) for visualization, and the numbers of colonies with diameters >1 mm were counted [5]. The survival percentage was expressed as relative seeding efficiency of UVC-irradiated versus mock-irradiated cultures.

### 3.4. Immunoblotting

Cells were harvested and washed twice in ice-cold PBS, and then lysed in RIPA buffer (1% Triton X-100, 20 mM Na_2_HPO_4_, 100 mM NaCl, 0.2 mM PMSF). Lysates were boiled in SDS sample buffer [62.5 mM Tris (pH 6.8), 5% β-mercaptoethanol (Merck, Darmstadt, Germany), 10% glycerol, 2% SDS, 0.001% bromophenol blue], and then were separated by 10% SDS-PAGE. The separated proteins in SDS-PAGE were then electro-transferred to Hybond-PVDF membrane (GE Healthcare). The PVDF membrane was then soaked in a blocking solution containing 5% (w/v) non-fat milk in TBST [20 mM Tris, pH 7.5, 0.5 M NaCl, 0.1% (v/v) Tween-20] for 1 h at room temperature. To assess COX-2 levels, the blocked PVDF membranes were then incubated with antibody against COX-2 [diluted 1:2000 in 5% (w/v) non-fat milk in TBST] at 4 °C overnight, and then washed with TBST three times for 15 min each and incubated in horse-radish peroxidase-conjugated goat anti-mouse IgG antibody (diluted 1:5000 in TBST buffer) at room temperature for 1 h. The membrane was washed three times for 15 min with TBST buffer. Immunobands were detected by enhanced chemiluminescence reaction (ECL, GE Healthcare). Equal loading was assessed by protein concentration determinations using Protein Assay kit (Bio-Red, Richmond, CA, USA) and by Coomassie blue staining of the gel.

### 3.5. Prostaglandin E2 (PGE2) Measurement

Cells were grown in 12-well plates overnight. 30 min before harvesting of culture media, the culture media of the cells were changed to new media, and then these culture media were centrifuged (600 × g, 3 min, at 4 °C) to remove cell debris. Cell-free culture media were collected at indicated times and PGE2 levels were determined by competitive enzyme-linked immunosorbent assay (ELISA) as described by the kit manufacturer (Cayman Chemical, Ann Arbor, MI, USA) using an ELISA reader (μQuant; Biotek Instruments, Inc, Winooski, VT, USA).

### 3.6. Cell Cycle Analysis

Cells (2 × 10^6^) were fixed in 70% ethanol (in PBS) on ice for 30 min and then resuspended in PBS containing 40 μg/mL propidium iodide and 0.1 mg/mL RNase (Roche, Mannheim, Germany). After incubating for 30 min at 37 °C, the fixed cells were analyzed by a flow cytometer (FASCSCalibur; Becton-Dickinson, San Jose, CA, USA) equipped with an argon-ion laser at 488 nm.

### 3.7. Apoptotic Cell Analysis

Control and UVC-irradiated NIH 3T3 cells and A431 cells were fixed by 70% ethanol overnight, and then were stained with 4′,6-diamidino-2-phenylindole (DAPI) solution (1 μg/mL DAPI, 0.1% triton X-100 in PBS) for 5 min at 37 °C. The percentage of UV-induced apoptotic cells with fragmented DNA (sub-G1 cells) were detected by flow cytometer (FASCSCalibur; Becton-Dickinson, San Jose, CA, USA) or image cytometer NucleoCounter NC-3000^TM^ (ChemoMetec, Denmark) [33].

### 3.8. Statistical Analysis

Data are expressed as means ± standard deviations (SD). Statistical significance was analyzed by Student’s *t* test for comparison with two groups and one way ANOVA for comparison with more than two groups. Differences resulting in *P* values < 0.05 were considered to be statistically significant.

## 4. Conclusions

UVC irradiation induces similar profiles of PGE2 secretion from NIH 3T3 cells and A431 cells, but induces different expressions of COX-2 in the two cell lines. Our data indicated that PGE2 is not absolutely suitable for representing the activity of COX-2 after UVC irradiation, and that COX-2 might play different roles in cellular response to UVC irradiation in various cell lines.

## Figures and Tables

**Table 1:** Changes in cell cycle of NIH 3T3 cells and A431 cells following exposure of UV.

The Cell Cycle Distribution after UV Irradiation in NIH 3T3 Cells					

Time	0 h	6 h	12 h	24 h	36 h

Cell Cycle					
% of G1 phase	63.50 ± 0.38	59.37 ± 1.54	38.86 ± 0.66 *	36.37 ± 0.60 *	28.95 ± 6.50 *
% of S phase	15.41 ± 2.0	17.00 ± 1.08	35.99 ± 0.60 *	37.36 ± 3.02 *	37.68 ± 1.08 *
% of G2/M phase	21.08 ± 1.77	23.62 ± 0.94	25.14 ± 1.16 *	26.01 ± 3.14	33.36 ± 7.37 *

**The Cell Cycle Distribution after UV Irradiation in A431 Cells**					

**Time**	0 h	6 h	12 h	24 h	36 h

**Cell Cycle**					

% of G1 phase	53.05 ± 3.47	60.13 ± 0.51 *	56.84 ± 2.66	53.22 ± 4.31	51.09 ± 2.25
% of S phase	22.93 ± 3.61	22.35 ± 1.91	24.64 ± 0.40	27.68 ± 0.82*	29.40 ± 1.80 *
% of G2/M phase	24.02 ± 0.20	17.52 ± 1.56	18.55 ± 3.04	19.10 ± 4.98	19.52 ± 4.02

Cells were irradiated with 20 J/m^2^ of UV, and were then harvested at the indicated times and cell cycle distribution analyzed by a flow cytometer. Data were from 3 independent experiments.

* *P* < 0.05, as compared with control cells which were not treated with UV irradiation at initial time (0 h).
